# The impact of IRT and IDT on metaverse adoption in tourism: The moderating role of personal innovativeness among China’s generation Z

**DOI:** 10.1371/journal.pone.0327239

**Published:** 2025-06-24

**Authors:** Weifang Zhan, Yaru Shi, Chengpeng Lin

**Affiliations:** 1 Department of Hotel & Convention, Honam University, Gwangju, Republic of Korea; 2 Department of Business Administration, Chonnam National University, Gwangju, Republic of Korea; Gachon University, KOREA, REPUBLIC OF

## Abstract

This study examines the Metaverse—a virtual world that merges entertainment, work, and social interactions—in terms of leisure tourism, using the frameworks of innovation resistance and diffusion theories. It explores the factors that drive and hinder Chinese Gen Z tourists’ resistance to Metaverse technology, including the moderating role of personal innovativeness. An analysis of data from 400 Chinese Gen Z tourists revealed that, in addition to risk barriers, factors such as value, usage, image, and traditional barriers significantly impact innovation resistance. Conversely, this resistance is mitigated by compatibility, trialability, and relative advantage, as outlined in the Innovation Diffusion Theory (IDT). Additionally, Innovation resistance significantly and negatively affects usage intentions, while personal innovativeness influences innovation barriers and diffusion within the Metaverse. This study not only fills gaps in existing studies but also provides valuable insights for providers and developers of Metaverse tourism services.

## Introduction

The Metaverse represents the biggest technological change of the 21st century in the tourism, hospitality, and leisure industries [1]. Propelled by the rapid advancement of the global digital economy, the Metaverse concept has swiftly transitioned from theoretical to tangible, emerging as a focal point in industry, business, and public domains. The Metaverse creates a comprehensive digital meta-space where users can work, play, and interact using digital avatars and Metaverse technologies including AR, VR and XR [2,3]. As technology progresses and travelers choose to stay home due to the coronavirus pandemic, Metaverse tourism is emerging as a viable alternative. It offers immersive virtual experiences that substitute physical travel and introduce new modes of tourist engagement. Although it presents a novel tourism form that differs significantly from traditional methods without requiring physical presence at attractions, many Metaverse tourism products remain nascent due to their short development cycle.

Metaverse has gained fame over the past couple of years in China’s and the global tourism sector [3]. There is an increased number of destinations and businesses exploring this latest technology to offer different tourism experiences in addition to increasing new utilization of this technology. Simultaneously, even with such great potential to advance technologically, numerous barriers result in this technology becoming limited. The phases of acceptance and adoption may face user resistance to new technologies due to challenges such as technological complexity [4], privacy concerns, and investment costs. This is especially critical among the generation born after 1995, who are attracted to personalization and immersion. They are likely to show more interest in interactive and visually appealing digital content [5]. Though several studies have been done on the acceptance of new technologies among Generation Z [6], skepticism and resistance to the Metaverse are high due to its complexity and perceived risks. It is therefore important to investigate innovation barriers faced by China’s generation Z in the Metaverse and how personal innovativeness influences their usage intention.

Innovation Diffusion Theory (IDT), proposed by Rogers, examines the reasons, methods, and rates at which new ideas and technologies spread across cultures [7,8]. IDT is broadly applicable across various academic fields. Although numerous studies have applied IDT across different sectors, research related to innovation resistance remains insufficient [9]. The diffusion of new technologies or ideas, including revolutionary ones like the Metaverse, necessitates extensive testing to achieve stable acceptance, highlighting the importance of exploring both drivers of acceptance and factors increasing resistance.

Initial scholarly focus on the Metaverse emphasized its adoption and diffusion by applying frameworks like Value-Based Adoption Model and Technology Acceptance Model (TAM) [10,11], highlighting its potential benefits, user experience, and economic impact. Current studies mainly focus on the favorable impacts of Metaverse adoption and the drivers behind users’ adoption intentions [12]. However, not all innovations receive a positive reception from consumers; the disruptions they cause can trigger resistance to change and the innovation itself [13,14]. This study differentiates itself by exploring this negative aspect, addressing a gap in the literature. While IDT explains user acceptance, it does not account for non-adoption, which Innovation Resistance Theory (IRT) addresses by highlighting reasons for resistance. This research integrates IDT and IRT into a comprehensive framework to explain both adoption and resistance among China’s Generation Z.

Choubey et al. [8] employed both qualitative and quantitative methods to integrate IDT and IRT, focusing only on technologically adept graduates and professionals, which limits the generalizability of their findings and overlooks broader psychological barriers. This study empirically examines Generation Z tourists in China, expanding the framework to include personal innovativeness and further exploring the dissemination and acceptance of the Metaverse.

This study constructs a comprehensive theoretical framework by integrating IRT, IDT, and personal innovativeness. This approach enhances our understanding of Generation Z tourists’ behaviors in China within the Metaverse, addresses limitations identified in previous studies, broadens the scope of current research, and offers insights for future investigations into the diffusion and acceptance of innovative technologies. It also provides essential guidance on how Metaverse tourism service providers and technology developers can more effectively develop and utilize Metaverse technologies to serve tourists, providing practical insights for effectively promoting these technologies.

Although existing research has combined IRT and IDT to explore the adoption and resistance of Metaverse technologies in tourism, it remains unclear whether personal innovativeness plays a moderating role. Furthermore, focusing on China’s Generation Z is still limited. Therefore, this research explores the research questions outlined below: What are the innovation barriers encountered in the utilization of the Metaverse? How does the IDT influence resistance to innovation and usage intention? How does personal innovativeness moderate the effects of innovation barriers on innovation resistance and usage intention?

The subsequent sections provide a summary of the relevant literature, describe the research methods, present the results, and discuss the results, limitations, future directions and conclusions.

## Literature review

### China’s generation Z in metaverse tourism

Neal Stephenson introduced the term “Metaverse” in his 1992 book, *Snow Crash*. The term “metaverse” combines “meta” (meaning “beyond”) and “verse” (meaning “universe”) [15]. Since its inception, the Metaverse has been described as a collective digital space with three-dimensional graphics accessible via screens or virtual reality technology [16 p18]. This space enables people to use AR and VR technologies [17 p1]. Launched by Linden Lab (2003), Second Life actualizes this concept, offering a virtual platform where users can create digital avatars to pursue various activities and exchange digital assets [18]. The metaverse extends beyond a visualized digital world to a cognitive space that necessitates physical engagement. Embodied cognition theory posits that human thought is profoundly influenced by the body’s interactions with its environment, enhancing experiential understanding through sensory and physical contact with the landscape [19]. Embodiment encapsulates how individuals perceive and interpret the world through both mind and body [20]. During the COVID-19 pandemic, restrictions on physical movement and human contact spurred the popularity of the metaverse, which offered alternative embodied experiences, recently applied in the tourism sector [21].

This paradigm shift has spurred increased research interest in applying Metaverse technologies in the travel and tourism industry. The Metaverse is described by Dwivedi et al. [22] as a virtual, interconnected space that allows users to engage in immersive, digitally constructed environments that either mimic or expand upon reality. Ji et al. [23] describe the Metaverse as a giant interactive virtual environment, consisting of interconnected environments where users communicate with digital assets and other users, simulating real-life interactions. Metaverse technology facilitates virtual reality travel experiences, virtual tour guides, and virtual destination exploration. A key feature of the Metaverse concept is its focus on activities within virtual worlds.

In terms of Metaverse like AR, VR and blockchain technologies, China is emerging as a global leader [24]. Simultaneously, China has the biggest domestic tourism market in the world, and the Chinese government is promoting the digital economy and driving technological innovation. These factors create significant potential for the growth of Metaverse tourism. A new demographic attracted to the Metaverse, and its digital experiences includes Generation Z, born after 1995 and raised in the digital world [25]. They seek dynamic, unique experiences, prefer adventure, and value living in the moment [26,27]. Ozdemir-Guzel and Bas [25] note that the tourism sector is particularly focused on this key generation that uses smart devices and applications for nearly all activities, resulting in a greater inclination towards virtual experiences offered by the Metaverse.

Current studies indicate that adopting Metaverse technology in the travel sector holds considerable potential for enhancing user experience and engagement. Future research could benefit from exploring the motivations and preferences of different user demographics, such as Generations Z and Y, regarding Metaverse tourism [28]. However, these studies are limited as they primarily examine factors influencing the willingness to engage with the Metaverse [15]. Rapid changes and advanced technologies can also provoke resistance among users [29]. So, it will influence the changes in the leisure or travel intentions of Generation Z. Addressing these challenges is vital to establishing trust and securing long-term adoption of Metaverse-based tourism.

### Innovation diffusion theory (IDT)

IDT models the entire pattern of diffusion, evaluation, and acceptance of innovative technology or services in society [30,31]. Rogers [32] describes an innovation as a new idea, practice, or item perceived by adopters and explains diffusion as the process by which an innovation evolves and spreads among participants in a social network via designated pathway. Factors such as observability, trialability, compatibility, complexity, and relative advantage of new technologies influence consumer acceptance [33]. Choubey et al. [8] applied IDT to the Metaverse, identifying three consistently linked factors to innovations: relative advantage, trialability, and compatibility. This study will also utilize these three IDT variables. In studies on the Metaverse and emerging technologies, these variables are outlined below: Compatibility refers to the ease of use for users [8]. Trialability allows an innovation to be tested before acceptance or rejection [30]. Relative advantage measures the perceived benefits of an innovation over previously used options [34]. Although IDT is a useful framework for studying individual technology acceptance behavior, it has been criticized for not detailing the adoption processes and for solely focusing on factors influencing acceptance [35]. To address these limitations, IDT is often used in combination with other theoretical models to study individual acceptance of innovative technologies.

### Innovation resistance theory (IRT)

The IRT model was first introduced by Ram & Sheth in 1989, who outlined that innovation resistance characteristics include timing, the degree of product difference from previous products, and product classes. This was further refined by Heidenreich and Spieth [36], and Talke and Heidenreich [37], who emphasized that it addresses the physiological and psychological resistance of customers to innovative products. According to IRT, customers can resist both actively and passively: Customer active resistance stems from conscious associations with novelty features. It is about functionality barriers in terms of usage, value, and risk. On the contrary, passive resistance is the result of an incongruence with what the consumers had previously perceived; it is driven by traditional and image barriers. The inability to use or the unfamiliarity with the innovation is called the usage barrier [8]. The value barrier signifies the expected advantages and economic value, especially the performance-to-price ratio that the innovation fails to provide compared to its alternatives [38]. Risk barriers are hesitations to adopt an innovation due to uncertainties about its outcomes [8]. Tradition barriers are obstacles that arise when an innovation requires changing existing habits, cultures, and behaviors of users [39]. Image barriers are defined as negative impressions of an innovation that center on perceptions of either complexity or origin [40].

Resistance to innovation concerns the technological or psychological refusal of the very innovative technologies by their users and is considered a big barrier in their adoption [14,41,42]. In this context, in the case of the Metaverse, this could be linked to psychological reasons such as fear of technology, perceived risks, or even lack of confidence in the platform [14]. Talwar et al. [43] defined innovation resistance as “the disposition of consumers to resist the adoption of new technologies or services due to perceived barriers,” which may include both functional and psychological elements. Kumar et al. [44] innovation resistance as the reluctance or opposition of individuals and organizations to embrace new technologies.

### Usage intention (UI)

Individuals’ usage intention is employed as a positive response variable across various research domains to predict future consumer behavior—the stronger the intention to use, the more positive the perception becomes regarding the offering [45,46]. Considered essential for forecasting consumer behavior (the planned execution of specific objectives) and has become a significant variable in consumer behavior studies [47,48].

Park and Kang [11] define the usage intention in the Metaverse as the attitude of readiness or intention in users to use the technologies of the Metaverse. Lee and Kim [15] describe usage intention as users’ intent to use the Metaverse platform based on factors from the UTAUT. Zarouali [49] defines usage intentions as the disposition or tendency that people must engage with Metaverse technologies related to perceived gratifications that will satisfy entertainment, social interaction, and information acquisition.

### Personal innovativeness (PI)

Personal innovativeness is one’s tendency towards new stimuli and an increased propensity to try new products or technologies ahead of others in their social system [50]. Rogers [50] categorized customers into four groups based on personal innovativeness: (1) late majority (i.e., laggards) (2) early majority, (3) early adopters, (4) innovators. Armstrong et al. [51] defines innovativeness as a consumer’s disposition to take risks associated with using technology. The level of innovativeness consumers exhibit has significantly affected both the rate of adoption of innovations and resistance to innovations, playing a crucial role in assessing their acceptance of new technologies [52,53]. In the Metaverse, personal innovativeness, as explained by Handoko et al. [54], describes “a user’s belief in one’s capability to match or adopt new technologies or innovations.” Sowmya et al. [55] describe personal innovativeness as “a generalized tendency among individuals to adopt an idea or technology more quickly than others.”

## Theoretical background and hypothesis development

### IRT and innovation resistance

In the context of the Metaverse, innovation resistance has been examined. Joachim et al. [56] investigated “active innovation resistance” by examining psychological and functional barriers in various settings. The results indicated that both types of barriers significantly affect innovation resistance. Talwar et al. [43] explored deterrents to purchasing through digital booking platforms and found that risk-related and value-related barriers lead to consumer resistance. Furthermore, Sowmya et al. [55] developed a model based on IRT to analyze the barriers tourists face when adopting the Metaverse and identified major factors contributing to resistance. The study shows that functional and psychological barriers are significant deterrents to tourists’ readiness to engage with Metaverse experiences.

Based on the above studies, the literature on innovation resistance in the Metaverse confirms that identifying and addressing perceived barriers to this transformative technology is crucial, as these barriers impact resistance. Graded through IRT in this way, researchers can come up with strategies to reduce resistance and implement the metaverse into everyday tourism successfully. Consequently, building on existing research, we have proposed the following hypotheses:

H_1_: Perceived innovation barriers in Metaverse tourism are positively associated with innovation resistance.

H_1a_: Perceived usage barriers in Metaverse tourism are positively associated with innovation resistance.

H_1b_: Perceived value barriers in Metaverse tourism are positively associated with innovation resistance.

H_1c_: Perceived risk barriers in Metaverse tourism are positively associated with innovation resistance.

H_1d_: Perceived traditional barriers in Metaverse tourism are positively associated with innovation resistance.

H_1e_: Perceived image barriers in Metaverse tourism are positively associated with innovation resistance.

### IDT and innovation resistance

Research on innovation resistance in IT services mainly focuses on the use of TAM and IDT. Scholars have subsequently integrated various theories and models for further exploration. For instance, Yun [57] combined the core constructs of TAM, IDT, and IRT to assess the dominant drivers of television users’ continuance intentions. It was found that, aside from the factor of relative advantage, all other variables had statistically significant positive impacts on innovation resistance behavior. In addition, complexity and compatibility were identified as significant barriers to innovation adoption in a study by Lee [58] among TV users. The IDT Model, IRT Model, and perceived risk theory were applied to online banking users regarding intention to accept innovations by Bae [59]. When applicability, trialability, and observability are high and complexity is low, acceptance of innovation tends to be higher; conversely, resistance to innovation is more likely [60,61]. Although these studies integrate various models for research, they do not examine the fundamental barriers to forming innovation resistance models. Addressing this gap, Choubey et al. [8] employed the theoretical frameworks of IDT and IRT to investigate the fundamental barriers in developing an innovation resistance model, providing an in-depth analysis of tourists’ usage intentions in the Metaverse. As a result, based on existing research, we have proposed the following hypotheses:

H_2_: IDT factors have a negative impact on innovation resistance in the Metaverse.

H_2a_: Compatibility is negatively correlated with innovation resistance.

H_2b_: Trialability is negatively correlated with innovation resistance.

H_2c_: Relative advantage is negatively correlated with innovation resistance.

### Innovation resistance and usage intention

Innovation resistance is widely recognized as a key element for the introduction and acceptance of new media or technologies, with several studies confirming that higher innovation resistance among social members regarding new media or technology correlates with a lower intention to use such technologies [14,58,62]. Resistance can intensify if consumers act or oppose the adoption of innovation [63]. Zhang et al. [64] examined innovation resistance in relation to face recognition payment systems and observed a detrimental impact on users’ intentions to adopt these systems. Al-Adwan [65] conducted the inaugural study on consumers’ non-adoption intentions in meta-commerce using the IRT, finding that functional barriers significantly affect consumers’ intention not to adopt meta-commerce. Balhareth et al. [66] also examined the adoption of meta-commerce from a consumer perspective. By extending the TAM to include additional variables, they discovered that risk barriers negatively impact consumers’ intention to engage with meta-commerce. Consequently, based on existing research, the following hypothesis has been developed:

H_3_: Innovation resistance is negatively correlated with usage intention in the Metaverse.

### Moderating role of personal innovativeness

Personal innovativeness plays a crucial moderating role in innovation resistance. Rogers [50] suggests that the improvement of personal innovativeness increases risk-taking, technical understanding, and ability to cope with uncertainty, thereby reducing innovation resistance and increasing the likelihood of accepting it. Ju and Lee [67] found that those with greater personal innovativeness exhibit less resistance to adopting smart clothing. Similarly, Khalil et al. [68] showed that higher personal innovativeness reduces resistance to drone food delivery services, acting as a buffer against adoption barriers. Al-Adwan et al. [69] analyzed the adoption of meta-commerce from multiple perspectives by integrating the UTAUT2 model and Dual-factor Theory. They not only identified potential barriers to meta-commerce adoption but also confirmed that consumers’ innovativeness significantly influences their adoption intentions. In tourism, Sowmya et al. [55] determined that personal innovativeness moderates’ barriers to Metaverse adoption, with higher innovativeness reducing resistance. IDT also supports the moderating influence of consumer innovativeness. As a result, based on existing research, we have proposed the following hypotheses:

H_4_: Personal innovativeness moderates the influence of innovation barriers and innovation diffusion on innovation resistance in the Metaverse.

H_4a_: Personal innovativeness moderates the relationship between innovation barriers and innovation resistance in the Metaverse, such that higher personal innovativeness weakens the positive impact of innovation barriers on innovation resistance.

H_4b_: Personal innovativeness moderates the relationship between IDT factors (e.g., relative advantage, compatibility, trialability) and innovation resistance, enhancing the negative impact of these factors on innovation resistance.

**Fig 1:**
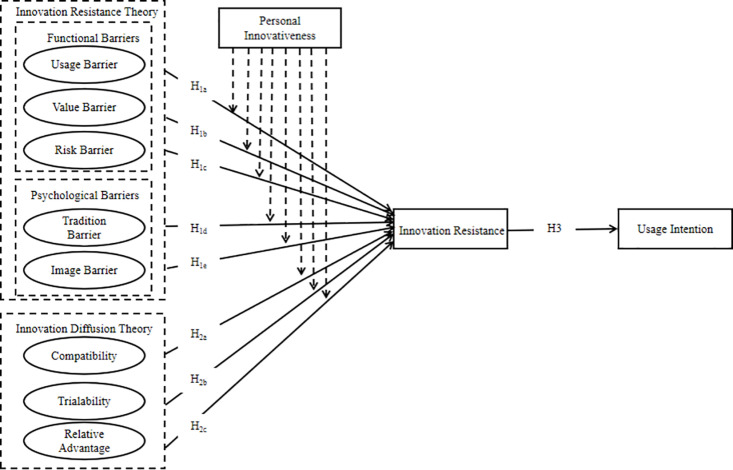
Conceptual model.

Summarizing all the above hypotheses, the conceptual model is shown in Fig 1.

## Methodology

### Survey instrument

To validate our hypothesis, we performed an online survey using Wenjuanxing, a professional online questionnaire platform in China. This self-administered survey method provided respondents with a link or QR code, allowing them to complete the questionnaire independently from October 1, 2024 to November 1, 2024. The questionnaire comprised of three sections: an introduction explaining the study’s purpose, scales adapted from existing literature, and demographic information. We defined “Metaverse” based on an extensive literature review as a shared digital space that integrates VR, AR, and MR technologies to offer immersive tourism experiences without physical movement [8,55]. Before participating in the survey, all participants were informed about the study’s purpose, voluntary nature, and their right to withdraw at any time without penalty. Participants provided informed consent by checking a consent box on the platform before proceeding to the questionnaire. This process ensured that participants’ involvement was voluntary and based on their understanding of the study.

All the scales for the constructs of innovation barriers, innovation diffusion, innovation resistance, usage intentions, and personal innovativeness were adapted from existing literature. Scale items were refined to be more relevant for this study. Innovation barriers were assessed using the scale of Sowmya et al. [55], with items such as “Metaverse-based tourism is more complicated” and “I perceive using the metaverse as riskier in tourism.” Innovation diffusion was assessed using the scale from Choubey et al. [8], including statements such as “Using the Metaverse to self-plan my tours would be compatible with my lifestyle” and “I would be able to test the suitability of the services.” Resistance to innovation was gauged with the items taken from the scale suggested by Al-Adwan [65], “I’m resistant to using Metaverse services” and “I feel dissatisfied with the use of Metaverse services.” Usage intention is derived from the scales by Kaur et al. [70] represented as follows: “I will always give Metaverse services in tourism a try.” and “If given the chance, I will use Metaverse services in tourism.” Personal innovativeness was adopted from Sowmya et al. [55]. Some items on the measure included “I always look for a chance to try new technology when I hear about it.” and “I enjoy trying out new tech products.” Subjects were asked to indicate agreement or disagreement based on their experience with five-point Likert scales from 1, which corresponded to “strongly disagree,” to 5, for “strongly agree”.

The final section of the questionnaire gathered demographic data, including gender, marital status, occupation, monthly salary, and educational background. To align the sample population with the research objectives, this study employed judgment sampling, specifically targeting Generation Z tourists in China, born between 1995 and 2009. This demographic is familiar with digital technology from a young age and adept at utilizing the Metaverse for tourism. To guarantee the reliability and accuracy of the findings, the research focused on a quantitative analysis of Chinese Generation Z individuals who have engaged with Metaverse-related technologies or applications during their travels within the past six months. Additionally, to enhance clarity and comprehension, the survey was translated into Mandarin.

### Respondents’ demographic profile

In total, we collected 450 complete questionnaires. To ensure response validity, 50 questionnaires that were incomplete or duplicated were excluded, and only questionnaires with a completion time of at least 5 minutes were included, resulting in 400 valid responses for statistical analysis. Statistical analysis was conducted with IBM SPSS 27 and IBM AMOS 26. The analysis process was structured as follows: (1) frequency analysis, (2) reliability and validity testing, (3) confirmatory factor analysis (CFA), (4) discriminant validity, (5) multicollinearity test, (6) path analysis, and (7) simple moderation analysis. A descriptive analysis of respondent demographics revealed a relatively balanced gender distribution, with females comprising 52.8% and males 47.3% of the sample. Students represented most participants, accounting for 53%. Additionally, most respondents were either undergraduate students or degree holders (Table 1).

**Table 1:** Socio-demographics of respondents (n = 400).

Profile	Frequency	Percent(%)
Sex		
Male	189	47.3
Female	211	52.8
Marriage status		
Married	118	29.5
Single	282	70.5
Profession		
Employee	89	22.3
Professional	43	10.8
Freelancer	21	5.3
Student	212	53
Unemployed	6	1.5
Other	29	7.2
Education level		
Junior high school graduate or less	8	2
High school graduation	15	3.8
Junior college enrollment/graduation	134	33.5
University enrollment/graduation	191	47.8
Postgraduate or above	52	13
Monthly income		
Less than RMB3000	170	42.5
RMB3000–5000	111	27.8
RMB6000–8000	77	19.3
RMB9000–14000	31	7.8
More than RMB 14000	11	2.8

## Results

### Common method bias test

Common method bias may occur when variations between variables are more influenced using the same research context, measurement tools, or data sources rather than by the actual relationships between constructs. To assess its potential impact, Harman’s one-factor test was performed in SPSS 27. With 12.284% of the total variance, the first factor was far below the 40% threshold, indicating that common method bias is unlikely to significantly compromise the study’s validity.

### Reliability and validity testing

We assessed the reliability of the scales using Cronbach’s alpha (Table 2). The Cronbach’s alpha coefficients for all dimensions exceeded the 0.7 threshold, confirming their sufficient reliability and internal consistency [71].

**Table 2:** EFA and CFA results.

Constructs	Statement	Label	Loading	Cronbach α	CR	AVE
Usage Barrier	Metaverse based tourism is more complicated.	UB1	0.631	0.972	0.921	0.972
	In my opinion immersive experiences using Metaverse are not much convenient.	UB2	0.612			
	I feel difficult to use Metaverse in tourism.	UB3	0.664			
Value Barrier	I feel Metaverse based tourism is more costly.	VB1	0.705	0.964	0.901	0.965
	The use of Metaverse tourism increased my spending.	VB2	0.718			
	I feel Metaverse experience doesn’t seem to have any advantages over other available alternatives.	VB3	0.629			
Risk Barrier	I feel using Metaverse is riskier in tourism.	RB1	0.747	0.943	0.850	0.944
	I feel that my personal credentials may be improperly used.	RB2	0.830			
	I think it’s easy to hack personal information with Metaverse.	RB3	0.828			
Tradition Barrier	When I use Metaverse, I miss the actual visit experience.	TB1	0.702	0.901	0.754	0.902
	I find it difficult to get accurate information about various places through the Metaverse experience.	TB2	0.698			
	From my point of view, the Metaverse services in tourism aren’t very good.	TB3	0.587			
Image Barrier	I feel that Metaverse to be too complex to use effectively.	IB1	0.711	0.985	0.970	0.985
	I think the Metaverse platform is hard to use.	IB2	0.721			
Compatibility	Using Metaverse to self-plan my tours would be compatible with my lifestyle.	C1	0.774	0.911	0.773	0.911
	Using Metaverse to explore unique destinations would be compatible with my preferences.	C2	0.746			
	Using Metaverse to self-plan my tours would be compatible with my needs and current situation.	C3	0.738			
Trialability	I can try using Metaverse for a while to explore its functionality.	T1	0.728	0.961	0.893	0.961
	I would be able to test the suitability of the services.	T2	0.716			
	I found it easy to try Metaverse.	T3	0.682			
Relative Advantage	Using Metaverse would comparatively improve my overall tourist experience.	RA1	0.548	0.991	0.981	0.990
	Using Metaverse would comparatively make it easier to plan tours.	RA2	0.558			
Innovation Resistance	I’m resistant to using Metaverse services.	IR1	0.511	0.976	0.931	0.976
	I hold a negative attitude towards using Metaverse services.	IR2	0.559			
	My experience with Metaverse services wasn’t great.	IR3	0.533			
Usage Intention	In the future, I plan to incorporate Metaverse services into my travel experience.	UI1	0.636	0.928	0.812	0.928
	If I have a chance, I will think about using the Metaverse for travel.	UI2	0.712			
	I will always give Metaverse services in tourism a try.	UI3	0.652			
Personnel Innovativeness	I always look for a chance to try new technology when I hear about it.	PI1	0.620	0.990	0.970	0.990
	I am sure I am ready and able to use new tech like the Metaverse.	PI2	0.639			
	I enjoy trying out new tech products.	PI3	0.615			

For validity, an exploratory factor analysis (EFA) was initially performed (Table 2). This analysis showed that the eleven dimensions accounted for a total variance of 92.412%, with Bartlett’s sphericity test of sphericity confirming statistical significance (p < 0.000; df = 465). The KMO value was 0.969, surpassing the 0.7 threshold and indicating the appropriateness of exploratory factor analysis. The variance explained cumulatively by each factor was as follows: Usage Barrier (12.316%), Value Barrier (24.370%), Risk Barrier (34.950%), Tradition Barrier (44.222%), Image Barrier (53.461%), Compatibility (62.479%), Trialability (70.087%), Relative Advantage (77.452%), Innovation Resistance (84.321%), Usage Intention (89.080%), and Personal Innovativeness (92.412%). These results demonstrate that each factor adequately captures the differences in the initial measurement items, providing initial support for measurement validity.

A CFA followed (Table 2). The CFA results show that the model fits well: χ2 = 523.927, df = 379, p < 0.000. The χ2/df = 1.382 is below the 3.0 threshold, suggesting an acceptable fit. The GFI is 0.926, above the threshold of 0.90. The CFI and TLI exceeded 0.95, at 0.993 and 0.991 respectively, indicating excellent fit. The RMSEA is 0.031 and the PCLOSE of 1.000, reflecting a very high model fit. The RMR is 0.017, indicating minimal residual error. These indicators confirm a strong alignment between the model and the data. Both discriminant and convergent validity were assessed with no concerns noted. All variables had CR values above 0.7, and AVE values approached or met 0.5. These results confirmed the strong construct convergence [72]. The AVE’s square root exceeded the off-diagonal inter-correlations, demonstrating the distinctiveness of the constructs [55] (Table 3). After confirming the reliability and validity, we performed a path analysis to test the relationship between hypotheses.

**Table 3:** Results of discriminant validity.

Variable	UB	VB	RB	TB	IB	C	T	RA	IR	UI	PI
UB	0.960										
VB	0.814	0.949									
RB	0.748	0.679	0.922								
TB	0.825	0.823	0.715	0.868							
IB	0.760	0.717	0.642	0.771	0.985						
C	−0.763	−0.798	−0.677	−0.768	−0.617	0.879					
T	−0.777	−0.764	−0.683	−0.839	−0.832	0.731	0.945				
RA	−0.828	−0.806	−0.701	−0.824	−0.731	0.825	0.794	0.991			
IR	0.843	0.838	0.708	0.867	0.812	−0.795	−0.853	−0.845	0.965		
UI	−0.833	−0.800	−0.694	−0.829	−0.752	0.780	0.800	0.831	−0.849	0.901	
PI	−0.837	−0.792	−0.732	−0.798	−0.776	0.749	0.784	0.824	−0.851	0.862	0.985

### Structural model

Structural equation modeling using AMOS revealed acceptable fit indices: χ2 = 494.720; df = 313; p < 0.000; χ2/df = 1.581; GFI = 0.920; CFI = 0.989; IFI = 0.989; RMR = 0.030; RMSEA = 0.038; PCLOSE = 0.000 [73]. Normally, when the VIF value is below 5, multicollinearity is not considered to significantly affect the model [74]. According to Table 4, the maximum VIF value was 4.724 (less than 5), indicating that multicollinearity does not compromise the robustness of the path analysis results. All hypotheses, except for H1c, were significantly supported (Table 4).

**Table 4:** Structural model results.

Hypothesis	Path	β	t-value	p-value	VIF	Results
H1a	UB → IR	0.132	2.815	**	4.541	Yes
H1b	VB → IR	0.152	3.119	**	3.982	Yes
H1c	RB → IR	−0.005	−0.155	n.s	2.413	No
H1d	TB → IR	0.214	3.068	**	3.711	Yes
H1e	IB → IR	0.183	4.149	***	3.467	Yes
H2a	C → IR	−0.135	−2.528	*	3.115	Yes
H2b	T → IR	−0.186	−3.331	***	4.255	Yes
H2c	RA → IR	−0.112	−2.559	*	4.724	Yes
H3	IR → UI	−0.863	−24.133	***	1.000	Yes

n.s = not supported, β = standardized regression weights, p = probability.

***p < 0.001, **p < 0.01, *p < 0.05.

### Moderating role of personal innovativeness

**Fig 2:**
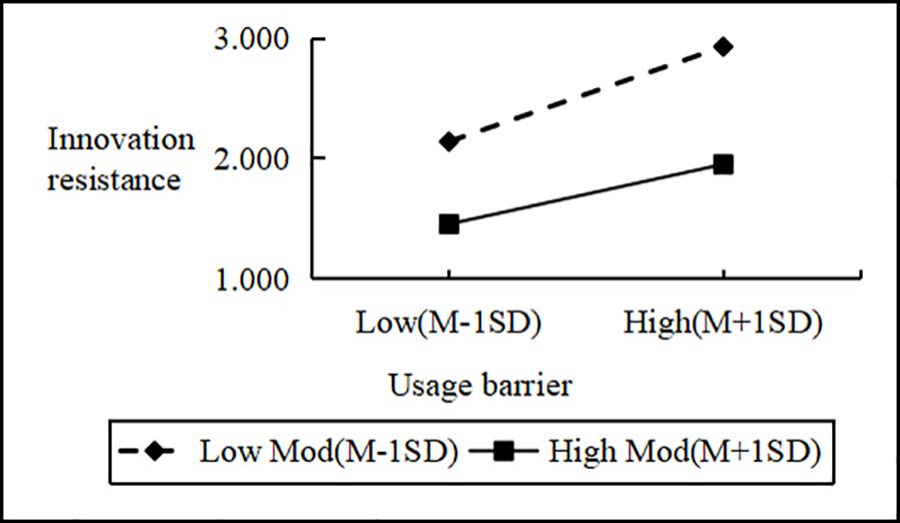
The moderating role of personal innovativeness on innovation resistance and usage barriers.

**Fig 3:**
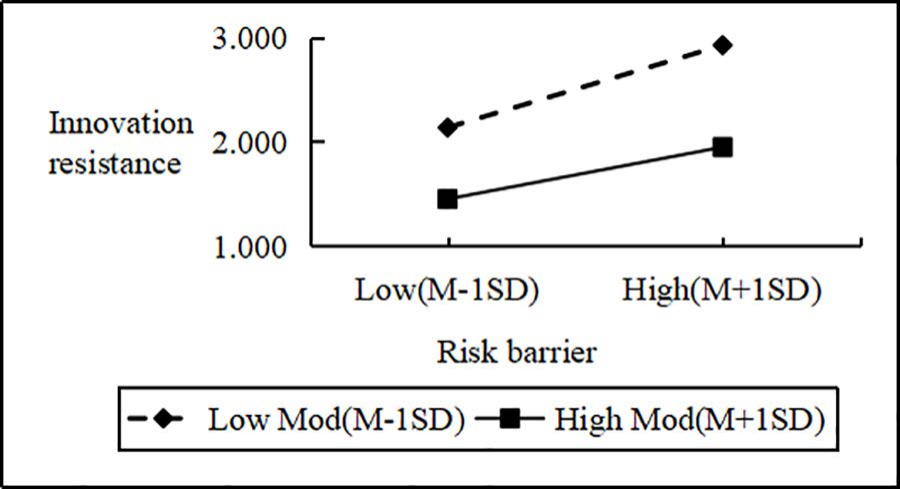
The moderating role of personal innovativeness on innovation resistance and risk barriers.

**Fig 4:**
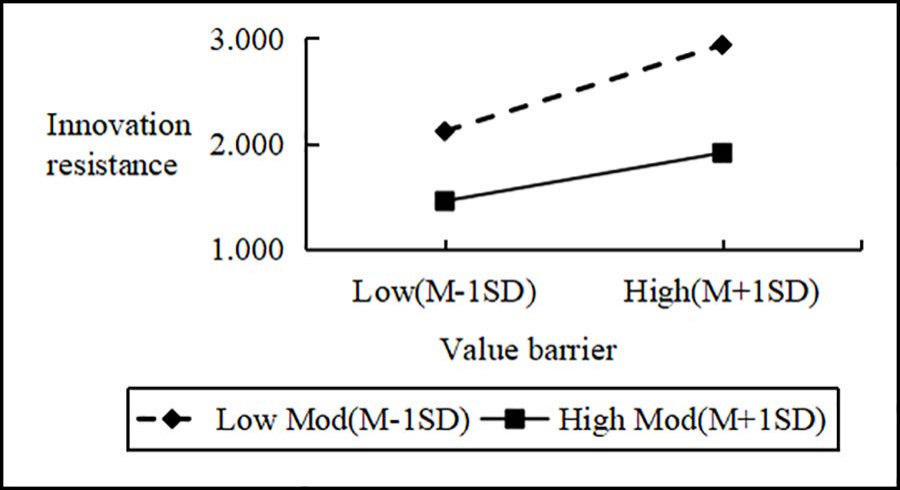
The moderating role of personal innovativeness on innovation resistance and value barriers.

**Fig 5:**
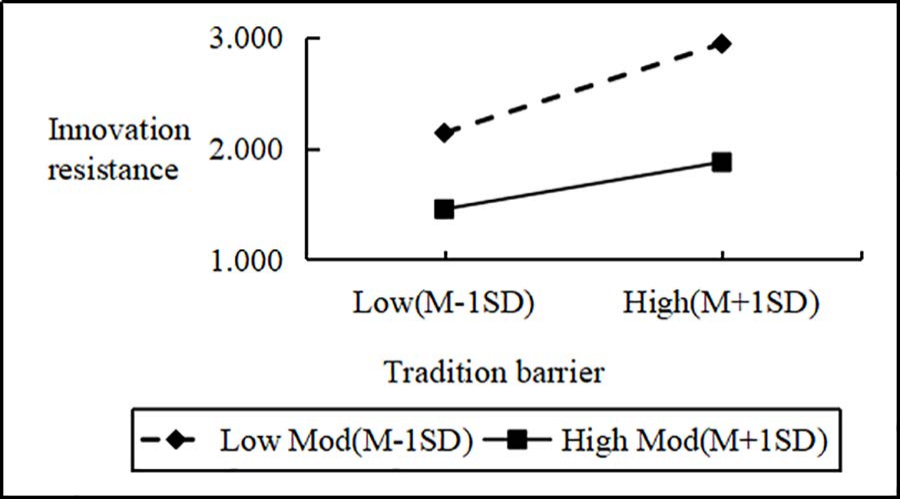
The moderating role of personal innovativeness on innovation resistance and tradition barriers.

**Fig 6:**
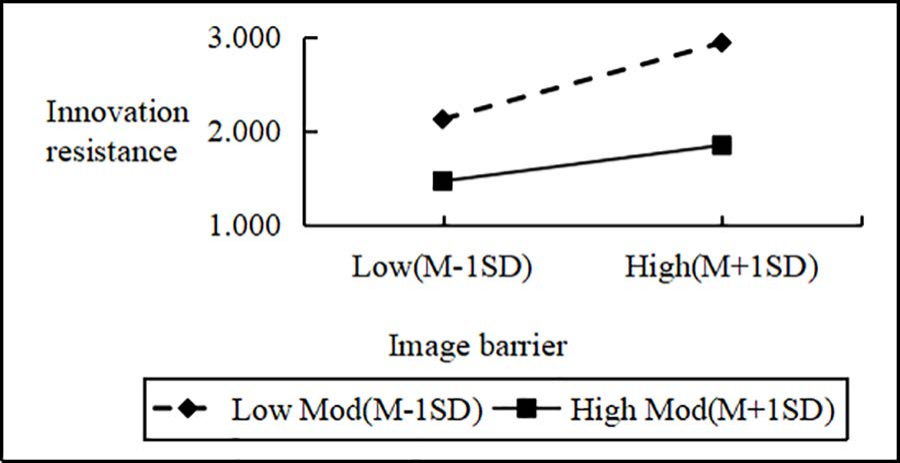
The moderating role of personal innovativeness on innovation resistance and image barriers.

**Fig 7:**
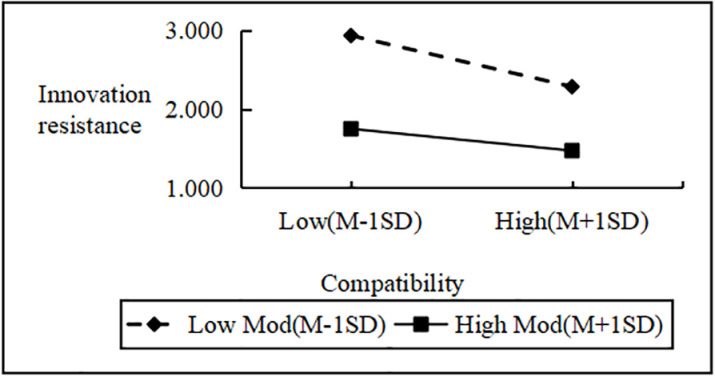
The moderating role of personal innovativeness on innovation resistance and compatibility.

**Fig 8:**
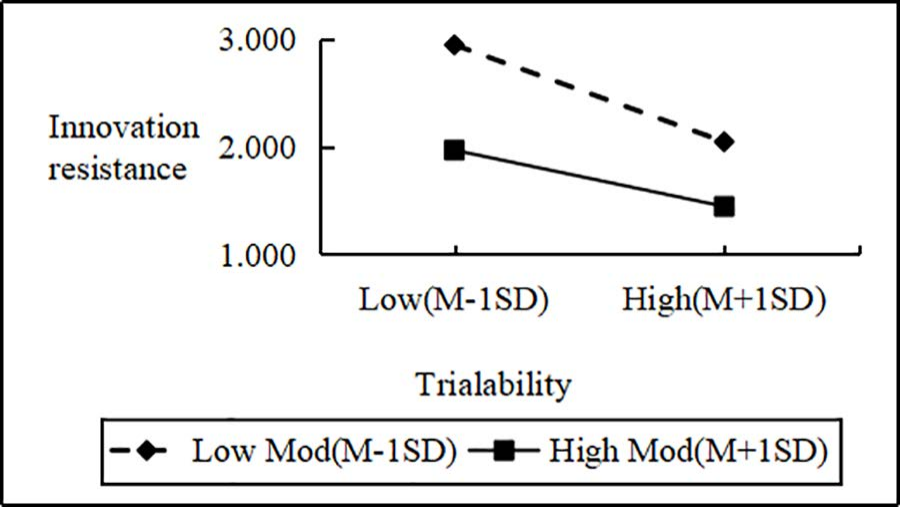
The moderating role of personal innovativeness on innovation resistance and trialability.

**Fig 9:**
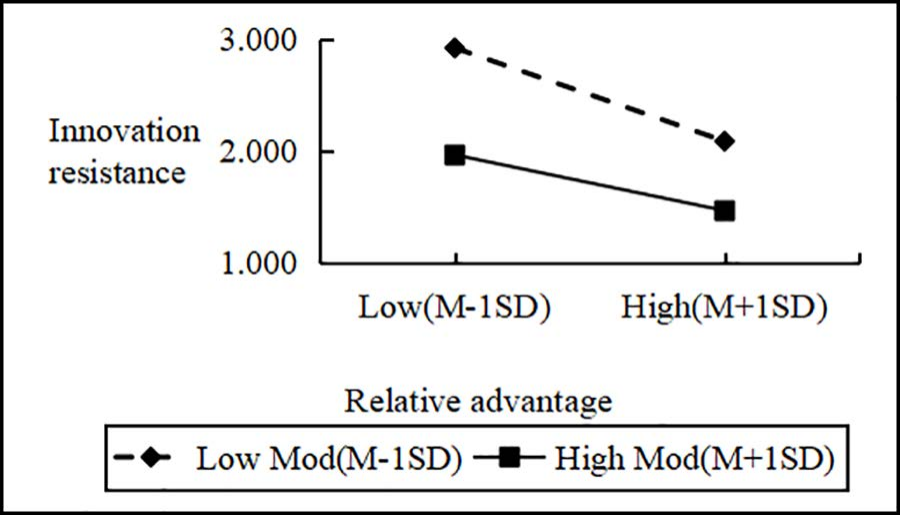
The moderating role of personal innovativeness on innovation resistance and relative advantage.

Regression analysis showed that Personal Innovativeness significantly moderates the relationships between various Innovation Barriers (e.g., Usage, Value, Risk, Tradition, Image) and Innovation Resistance. Interaction effects ranged from B = 2.090 to B = 2.114 (all p < 0.001). Including interaction terms significantly boosted the model’s explanatory power (ΔR^2^ = 0.751 to 0.797, all p < 0.001), confirming a moderating effect. Similarly, interactions between IDT factors (e.g., Compatibility, Trialability, Relative Advantage) and Personal Innovativeness significantly influenced Innovation Resistance (B values 2.102 to 2.108, all p < 0.001), improving model fit (ΔR^2^ = 0.776 to 0.806, all p < 0.001). These results support hypotheses H4a and H4b, indicating that higher Personal Innovativeness reduces the positive impact of Innovation Barriers on Innovation Resistance and enhances the negative impact of IDT factors on the same (Figs 2–9).

## Discussion

The results align with previous studies on innovation resistance in the Metaverse [43,56], which identified usage, value, traditional, and image barriers as significant factors positively associated with innovation resistance. Specifically, perceptions of complexity, high costs, deviation from traditional tourism practices, and negative attitudes toward new technologies were found to intensify resistance to Metaverse adoption in tourism. Among these, traditional and image barriers demonstrated particularly strong effects, highlighting the challenge of overcoming entrenched preferences for conventional tourism and negative perceptions of the Metaverse’s suitability. These findings contrast with recent studies on the adoption of meta-commerce [65,66,69], where functional barriers were found to negatively affect consumers’ intentions to adopt meta-commerce, consistent with our research findings. Functional barriers are a principal reason affecting the intention not to adopt the Metaverse in tourism among the Chinese Generation Z. This indicates that functional barriers are universal across different domains of Metaverse adoption. However, our study reveals that psychological barriers have a greater impact on the intention of Chinese Generation Z to resist metaverse technology in tourism. This underscores the significance of psychological barriers in the specific context of Metaverse tourism and provides crucial directions for enhancing Metaverse technologies, particularly by exploring barriers to adoption across different domains. Interestingly, risk barriers were found to be insignificant (H1c). This could be attributed to the technological orientation and high digital literacy of Generation Z in China, who have been immersed in digital environments from a young age [75]. Despite being aware of risks like data privacy concerns, they exhibit a higher tolerance for technological risks and a willingness to adopt Metaverse innovations.

The findings also reveal that the risk barrier (H1c) did not show a significant relationship with innovation resistance. This result may stem from the unique characteristics of China’s Generation Z, who have grown up in a digital environment. Their early exposure to the Internet and technology appears to have fostered higher tolerance for potential risks, such as data security and privacy concerns, as supported by Yi et al. [75]. Although Generation Z tourists perceive potential risks in adopting Metaverse technologies, their openness to innovation reduces its negative impact on adoption. Further analyses highlight that compatibility, trialability, and relative advantage are critical in reducing innovation resistance. Technologies that align with user needs, offer trial opportunities, and provide tangible benefits are more likely to lower resistance, aligning with Rogers’ [50] diffusion of innovation theory. For Generation Z, compatibility with their digital lifestyles, ease of testing, and visible advantages in tourism experiences significantly enhance their willingness to embrace the Metaverse. Moreover, this study confirmed that innovation resistance to Metaverse technology negatively influences usage intention. This supports earlier findings that higher resistance lowers the usage intention and decreases the possibility of adoption [58,64,65]. Addressing innovation resistance is thus crucial to promoting the adoption of Metaverse technology in the Chinese travel sector. We will discuss how these findings can help advance the growth and adoption of Metaverse technologies in the travel sector.

### Theoretical implications

This research integrates IRT and IDT to explore the utilization of Metaverse technology in tourism by Generation Z in China, expanding the scope of existing research. By overcoming limitations identified in previous studies [8], this research offers a more comprehensive framework that improves our understanding of innovation resistance and the diffusion of Metaverse technologies in tourism. In addition, this study extends IDT to the Metaverse, a field that has received limited attention, particularly in tourism [76].

Personal innovativeness as a moderating variable extends the theoretical discourse on individual differences in technology adoption. It demonstrates that young, tech-savvy individuals vary in openness and digital engagement due to personal traits. This emphasis on personal innovativeness supports the conclusions of Sowmya et al. [55] and provides empirical evidence of the interaction between personal innovativeness, perceived innovation barriers, and diffusion attributes in a newly emergent digital context.

The theoretical insights from this study refine the frameworks of resistance and adoption within digital tourism. Integrating these insights into a new comprehensive theoretical framework allows for holistic modeling of how digital technology is adopted and may interact with emerging and rapidly evolving technological landscapes like virtual reality, the Metaverse, and digital tourism. The integration of IRT and IDT broadens their applicability and lays the groundwork for further research on how these emerging interfaces affect digital tourism acceptance.

This research focuses on Generation Z from China, a highly digitally literate generation open to virtual experiences, highlighting the influence of sociocultural and generational contexts on Metaverse adoption. This behavior presents a solid basis for future studies on generational differences in the adoption of the Metaverse, informing tourism strategies for younger consumers.

### Managerial implications

This research provides valuable contributions for Metaverse tourism service providers and tech developers. First, we identified functional and psychological barriers limiting Metaverse adoption. To address these, providers must simplify technology operation processes, ensuring that users can easily start using the technology with minimal technical guidance. Moreover, the cost of using the technology should not exceed the typical travel budget, as high costs can lead to resistance and affect usage intentions. Additionally, privacy and data security are major concerns; thus, developers must prioritize data protection and maintain transparent privacy policies.

Second, this research underscores compatibility, trialability, and relative advantage as key IDT factors. Providers should align these elements with travelers’ preferences and lifestyles, adapting in real time to meet current needs and circumstances. In addition, providing tourists with opportunities to trial Metaverse technology and emphasizing its unique benefits can enhance its attractiveness over traditional tourism.

Third, personal innovativeness can moderate innovation resistance. Providers can foster tourists’ interest in new technologies by creating engaging games and activities that stimulate curiosity and encourage trials. Offering rewards for trying new experiences and building community platforms for feedback can promote interaction and cooperation among users and foster a sense of belonging.

Lastly, effectively activating the Metaverse tourism segment requires an understanding of the sociocultural and techno-economic characteristics of Chinese Generation Z tourists. While this generation is receptive to new technologies, they can also resist perceived innovation barriers. Therefore, providers should develop marketing strategies that emphasize the novelty of Metaverse technology and align with Chinese cultural values. Personalized campaigns and endorsements by influential Figs could further foster a receptive attitude towards Metaverse tourism.

### Limitations and future directions

This research presents meaningful insights into innovation resistance in Metaverse-based tourism; however, it also has several limitations that future research should address. First, the study is confined to Generation Z tourists in China, which limits its applicability to other regions and age groups. Future studies could extend to different countries and demographic groups to explore the effects of cultural and demographic variations on Metaverse adoption and resistance.

Second, while this research primarily utilizes IRT and IDT, incorporating additional models such as the TAM, the UTAUT, or the TPB could provide a deeper understanding of the drivers of Metaverse adoption in tourism. Finally, the research adopted a cross-sectional design; subsequent research could utilize longitudinal designs, such as time series analysis and longitudinal tracking, to better understand causality.

## Conclusion

This study combines IRT and IDT to examine the innovation resistance and usage intentions of Generation Z in China toward using Metaverse technologies in tourism, along with the moderating effect of personal innovativeness. The findings support most hypotheses, offering new insights into the utilization of Metaverse technology in China’s travel sector. Furthermore, our study indicates that personal innovativeness plays a moderating role by weakening the impact of innovation barriers on increasing innovation resistance, and strengthening the positive impact of IDT factors in reducing resistance. This finding suggests that Chinese Generation Z tourists with greater personal innovativeness have a propensity to adopt aspects related to innovation diffusion, such as compatibility, trialability, and relative advantage, and are less deterred by innovation barriers. Hence, personal innovativeness is crucial in adopting new technologies [55]. In conclusion, this research presents insights into the barriers to adoption of Metaverse technologies and the process to reduce resistance behavior. It imparts insight into the dynamics of innovation resistance in Metaverse tourism and offers practical implications for tourism service providers and technology developers. However, there are limitations, including the sample being limited to Chinese Generation Z tourists, the lack of integration of other models for a more comprehensive analysis of adoption drivers, and the use of a cross-sectional design. Future research should address these limitations by expanding the scope and diversity of the sample, integrating multiple theoretical models to analyze user acceptance of Metaverse technologies and behavioral intentions, and adopting a longitudinal design, which will ultimately contribute to overcoming barriers in the Metaverse adoption process, reducing resistance behaviors, and increasing users’ intentions to adopt Metaverse technology.

## Supporting information

S1 File Data. (XLSX)
