# Correction: The impact of IRT and IDT on metaverse adoption in tourism: The moderating role of personal innovativeness among China’s generation Z

**DOI:** 10.1371/journal.pone.0362101

**Published:** 2026-11-03

**Authors:** Weifang Zhan, Yaru Shi, Chengpeng Lin

The first two authors, Weifang Zhan and Yaru Shi, should be noted as shared first co-authors on this work.
